# The determination and role of peroxyacetil nitrate in photochemical processes in atmosphere

**DOI:** 10.1186/1752-153X-6-S2-S8

**Published:** 2012-05-02

**Authors:** Karim Movassaghi, Mario Vincenzo Russo, Pasquale Avino

**Affiliations:** 1 Department of Chemistry, University of Isfahan, Isfahan, 81744, Iran; 2 Facoltà di Agraria, Università del Molise, via De Sanctis, Campobasso, Italy; 3 DIPIA-ISPESL, via Urbana 167, Rome, 00184, Italy

## Abstract

Peroxyacetilnitrates (PAN) is the most characteristic photoxidant of a range of secondary pollutants formed by the photochemical reaction of hydrocarbons with nitrogen oxides in the atmosphere: it is phytotoxic and shows an increasing role in human health effects due to ambient air exposure, especially in presence of high ozone concentrations. Because of the similarity of the conditions required for their photochemical production PAN is observed in conjunction with elevated ozone concentrations. PAN has very low natural background concentrations so it is the very specific indicator of anthropogenic photochemical air pollution. In this paper we report PAN concentrations determined in Rome urban area during winter- and summer-period. PAN measurements were carried out by means of a gas-chromatograph equipped with an Electron Capture Detector (ECD) detector. For identifying the acute episodes of atmospheric photochemical pollutants the relationship between PAN and the variable O_x_ (=NO_2_+O_3_) which describes the oxidation process evolution is investigated. The role of Volatile Organic Compounds and PAN in the ozone formation is investigated as well the issue of taking in account the autovehicular emissions for checking the NO_x_ fraction in fuel.

## Background

Peroxyacetilnitrate (PAN, CH_3_C(O)OONO_2_) is the principal member of a family of nitrogenous compounds produced by action of sunlight on NO_x_ and reactive hydrocarbons [1]. PAN has been known to be a phytotoxicant [2,3] and lachrymator [3,4]. There has also been considerations with regard to the role of PAN in the human health effects due to the exposure in ambient air, especially in the presence of high levels of ozone [5,6]. PAN is a suggested agent of skin cancer [7] in photochemically active areas and a possible bacterial mutagen [8,9].

From an atmospheric chemistry point of view, PAN and O_3_ are the two most important components of photochemical smog, a very complex phenomena. Because of quite similar conditions required for their photochemical production, PAN is observed in conjunction with elevated ozone concentrations but there are differences in the characteristics of these two compounds. Basically, photochemical production of PAN and O_3_ are very closely linked as both initiated by the reaction of hydrocarbons with the hydroxyl radical (OH) and in presence of nitrogen oxides.

Although not well defined, the natural background concentration level of PAN is very low [3] so it is considered very specific indicator of anthropogenic photochemical air pollution; on the contrary ozone has relevant sources in stratosphere [10-12] where its level is high. It should be underlined that very few data are present in literature regarding of PAN levels in the atmosphere and so it is very hard to establish guideline values in air quality evaluation. Further, at low temperatures the PAN can represent an important reservoir for atmospheric odd nitrogen because the NO_2_ equilibrium (and the relative peroxyacetil radicals) depends strictly on the temperature [13]. Consequently, also the PAN lifetime in atmosphere strongly depends on the ambient temperature. This enables PAN to persist for a longer time at low temperature. Furthermore, PAN is slowly removed from the atmosphere through dry deposition; on the contrary, the ozone is rapidly removed and the dry deposition represents an effective destruction mechanism [14]. Therefore, episodes of long-range transport of PAN are likely to occur [15] and it is generally considered that PAN might constitute the largest fraction of the natural NO_x_ reservoir [14]: this is confirmed by recent observations of high PAN/NO_x_ ratios in the cool middle free troposphere [16].

Precursors of PAN in polluted areas are specific non-methane hydrocarbons (NMHCs) (particularly, propene, 1-butene, 2-butene, 2-pentene, etc.), aldehydes (formaldehyde, acetaldehyde) and NO_2_. Expecially in air masses polluted by anthropogenic emissions (i.e., autovehicular traffic and/or industrial emission), the NMHC abundance causes a rising of PAN mixing ratios sometime up to several ppbv [17].

In conjunction with the anthropogenic precursors, natural Volatile Organic Compounds (VOCs) such as isoprene [18] are relevant but of minor importance in urban and near urban atmospheres, where the PAN is the very specific indicator of anthropogenic photochemical air pollution [19-21].

**Figure 1:**
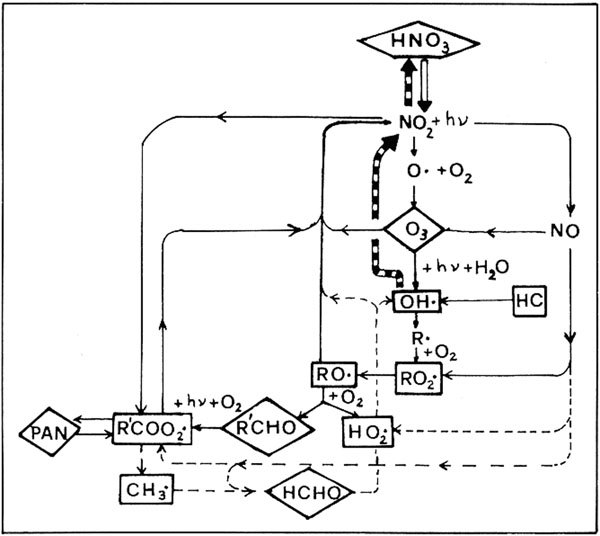
Master scheme of photochemical reactions occurring in atmosphere.

For identifying the occurrence of a strong photochemical smog episode in the atmosphere, the PAN, O_3_, NO_2_ and HCHO concentrations are measured and the variable O_x_ (sum of O_3_ and NO_2_) has been involved (Figure 1).

The present paper deals with the determination of PAN concentration in the urban area of Rome carried out in the period May 2007-April 2008. The meteorological conditions that may determine elevated PAN concentrations are here discussed and the relationships between measured concentrations of PAN, O_3_, NO_2_ and HCHO in this environment also described. A very important task of this research regards to set up the developing of an analytical procedure: indeed, the determination by a gas-chromatography technique (GC-ECD) does not require any sample enrichment. By means of this methodology the temporal evolution of these compounds can be followed and useful information on the photochemical pollution phenomena are derived and shown. Finally, it should be noted the difficulty to find out certified standard reference materials of PAN, Peroxymethylnitrate (PMN), Peroxypropionylnitrate (PPN) and CH_3_ONO_2_ for calibrating the instrumentations.

The reliability and accuracy of the analytical method have been verified through monitoring campaigns during photochemical smog episodes. Simultaneously, a large data-base on smog precursors (NO, reactive NMHCs) and the relative products (ozone, PAN, aldehydes) in Rome urban area was collected.

## Results and discussion

The monthly average PAN concentrations measured during the entire campaign are reported in Table 1. The PAN concentrations reached a maximum of 30.3 ppbv in summertime (average daily level of 5.7 ppbv) and a maximum of 7.3 ppbv in wintertime (average daily level of 2.1 ppbv).

**Table 1:** PAN levels (ppbv) determined in this study and relative comparison with other studies

*PAN level*	*Average value*	*Range*	*Reference*
Rome (this work)	2.1 (winter)	0.1-7.3	
	5.7 (summer)	0.1-30.3	
Antartica	9.3	0.8-33.2	[1]
Mexico City	15.0	0.1-34	[6]
Santiago	2.4	0.1-7.6	[3]

**Figure 2:**
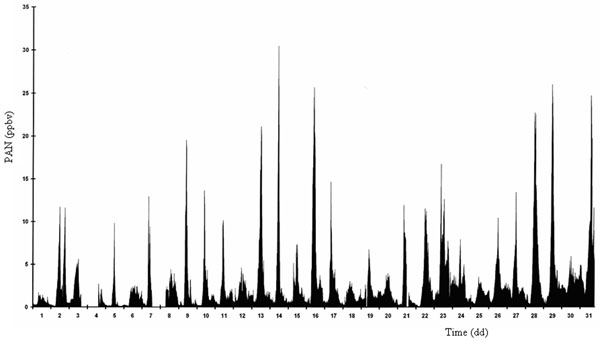
Typical daily trend of PAN during a summer period (Rome, Villa Ada).

**Figure 3:**
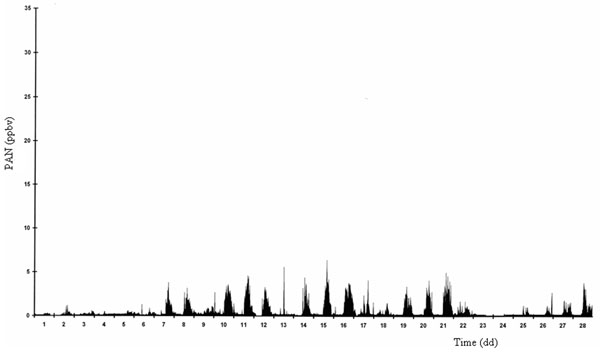
Typical daily trend of PAN during a winter period (Rome, Villa Ada).

In Figures 2 and 3 typical daily trends of PAN determined inside a green park, Villa Ada, in downtown Rome during summer and winter periods, respectively, are reported.

First of all, a clear difference about the amount is shown. During the summer period, the solar irradiation is strong and consequently the PAN production reaches very notable levels (up to 30 ppbv) compared with low levels in wintertime (maximum 5 ppbv). The really interesting consideration is that photochemical smog episodes occur also during cold period when the solar irradiation is low; meantime the VOC emissions are very significant as consequence of autovehicular traffic and domestic heating, characteristics of a great urban area such as Rome. Even if these episodes are limited and PAN does not reach high values, the occurring of this phenomena is important to understand the dynamics of the atmospheric pollution in the considered area and how the air quality is affected.

In Table 2 the average and max levels (expressed as ppbv) and the relative contributions (%) to the total amount are reported for each hydrocarbons C_2_-C_9_ determined in downtown Rome during the cold period.

**Table 2:** Average and max concentration levels (ppbv) and relative contribution (%) of each C_2_-C_9_ hydrocarbons measured in atmosphere of downtown Rome

*C_2_-C_9_*	*Average*	*Max*	*%*
Ethane	8.3	18.7	10.5
Ethene	20.4	126.0	25.7
Propane	6.0	25.6	7.6
Propene	3.3	14.4	4.2
n-Butane	4.1	32.0	1.3
i-Butane	1.1	27.8	5.2
1-Butene	1.1	10.1	1.3
cis-2-Butene	2.7	8.4	3.3
trans-2-Butene	0.2	4.3	0.3
n-Pentane	7.4	40.0	9.3
i-Pentane	2.0	84.9	2.5
cis-2-Pentene	0.5	5.5	0.6
trans-2-Pentene	0.7	5.5	0.9
2-Methylpentane	1.1	21.5	1.4
3-Methylpentane	3.6	22.2	4.5
Isoprene	0.2	1.6	0.2
n-Hexane	0.3	7.9	0.3
Cyclohexane	0.4	12.9	0.5
Benzene	3.5	3.0	1.3
n-Heptane	0.8	3.5	1.0
Toluene	5.9	20.4	7.9
Ethylbenzene	1.2	14.0	1.5
m- & p-Xylenes	3.9	16.5	5.0
o-Xylene	0.6	5.1	0.8
Styrene	1.2	6.1	1.5
1,2,4-Trimethylbenzene	0.6	3.4	0.8
1,3,5-Trimethylbenzene	0.5	3.5	0.6

*Total C_2_-C_9_*	*81.6*	*544.8*	*100.0*

The total composition (79.2 ppbv) is very complex and almost all the hydrocarbons in the range C_2_-C_9_ are present: in particular, alkanes 44.3%, alkenes 36.5% and aromatic 19.2%. Another interesting consideration is the high content of ethane (23.6%) and ethene (70.4%) to alkane and alkene fractions, respectively. Considering the origin of these two species [22,23], the values are ascribed to the strong diesel-vehicle density in downtown Rome.

As it can be seen in Figures 2 and 3, the PAN behavior is almost regular depending strictly on both the meteorological conditions and the ozone and HCHO levels in atmosphere, overall the VOC such as described above.

In fact, in presence of high concentration levels of VOCs, radicals RO_2_ and HO_2_ are formed according to the following reactions:(1)(2)(3)(4)

The radicals RO_2_ and HO_2_ react with NO giving NO_2_:(5)(6)

These last two reactions cause an increasing of the ratio NO_2_/NO and a relative ozone accumulation. At high NO_x_ concentrations such as those recorded in urban areas, the radicals RO_2_ and HO_2_ can be removed by other reactions giving formation of more stable compounds:(7)(8)(9)

**Figure 4:**
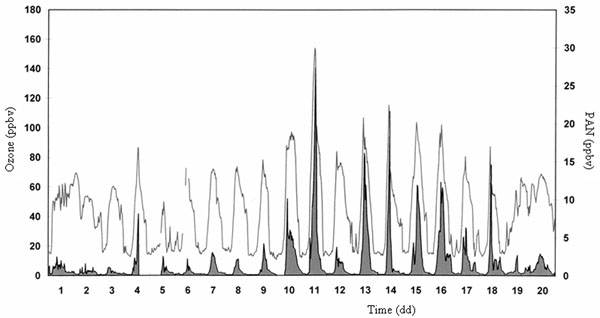
Typical daily trends of PAN (area) and ozone (line) during a summer period.

**Figure 5:**
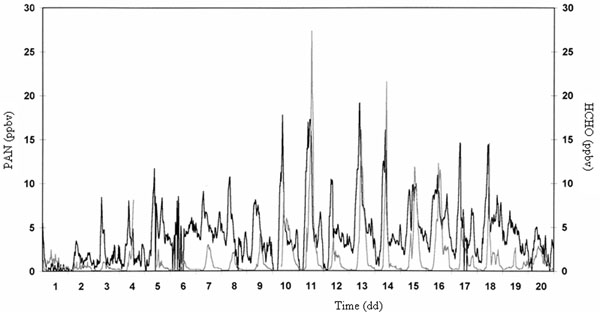
Typical daily trends of PAN (grey line) and HCHO (bold line) during a summer period.

**Figure 6:**
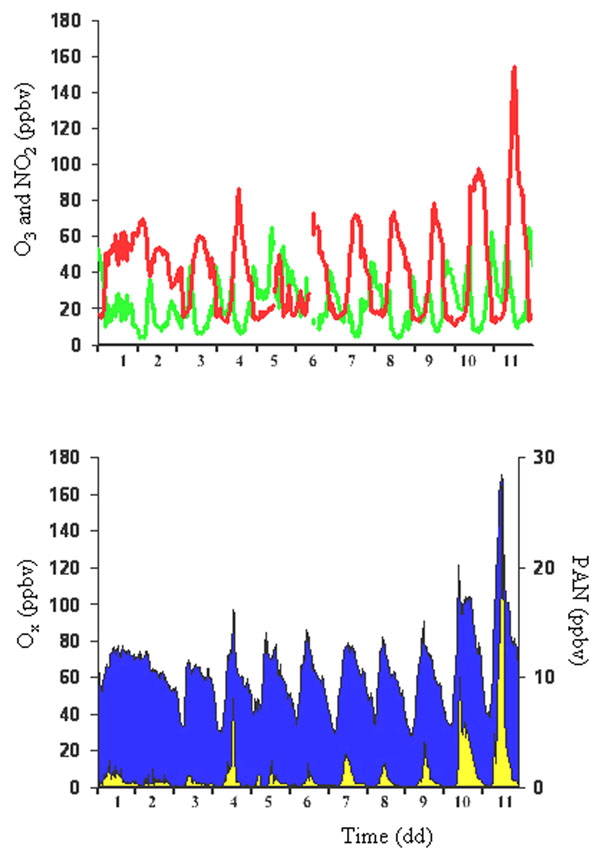
Typical daily trends of NO_2_ (green), O_3_ (red), O_x_ (blue) and PAN (orange) during a summer period.

In these condition the ozone formation kinetic is also influenced by other factors such as VOC species, the relative reaction coefficients for producing RO_2_ and OH radicals. It is well-known that nitrous acid (HNO_2_) and formaldehyde (HCHO) play a fundamental role in processes occurring in atmosphere [24,25]. In Figures 4-6 the trends of PAN, ozone, formaldehyde and O_x_ determined in downtown Rome, are reported.

First of all, looking at Figure 4 high correlation between PAN and ozone is found (Pearson’s coefficient of correlation 0.84) meaning a strict relationship between them. These considerations can be evident especially during stable atmospheric conditions (investigated using the natural radioactivity, radon) when the pollutant dispersion is not favored (from 7^th^ to 18^th^) whereas during the remaining periods a low correlation is observed which depends on the chemical reactions occurring in atmosphere.

Considering all the reactions above reported, Figure 5 shows that high HCHO concentrations are present in the late morning and afternoon whereas minimum values are found during high solar radiation hours: in particular, the higher levels are determined in the hour range 13.00-15.00 when the highest ozone concentrations are detected.

As it can be seen in Figure 6, the pollutant behaviors are interesting. In fact, the kinetics between PAN and HCHO are different: in atmosphere, the HCHO formation reaction is more rapid than the relative PAN formation whereas the PAN removal is very quickly. This means that during regular atmospheric mixing conditions (unstable conditions) no pollutant accumulation is possible: on the contrary, during stable conditions (i.e., when pollutant dispersion is not favored) smog photochemical episodes can occur. The intensity of such phenomena depends on variables above described: in any case the result of the event is very high PAN and HCHO levels and consequently maximum ozone concentrations.

**Figure 7:**
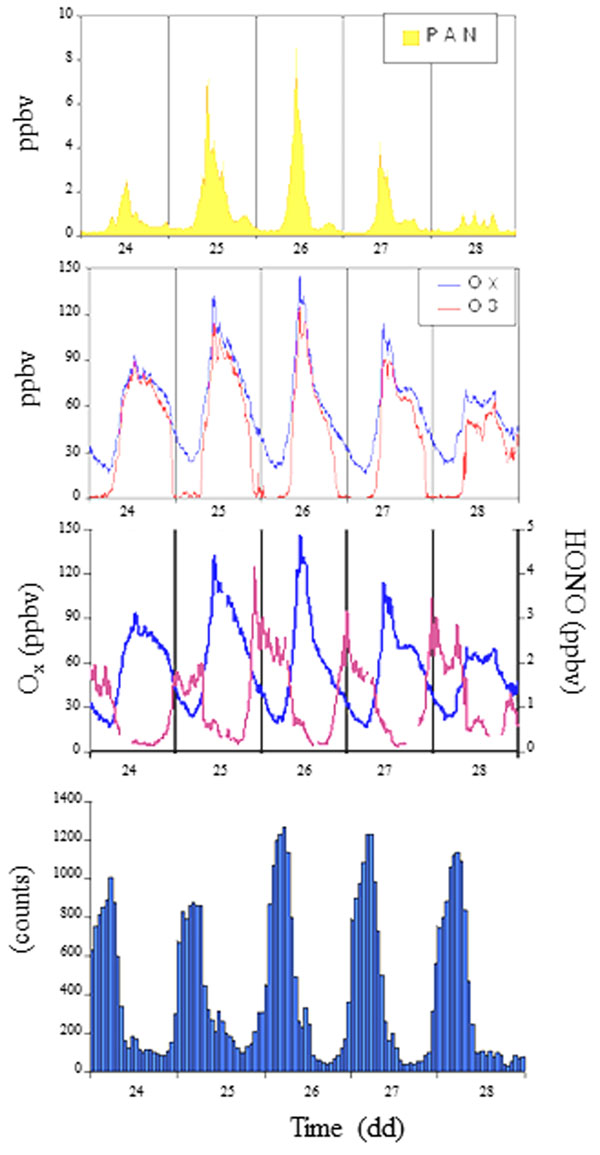
a) Typical trends of PAN, O_x_ and O_3_ during a summer period (June, 24^th^-28^th^) in relationship with the radon concentration behavior; b) trends of HNO_2_ and O_x_ during photochemical activity in summer period.

This different behavior is well-reported in Figure 6. The O_x_ variable is the sum of O_3_ and NO_2_ and describes the atmospheric radical conditions: when no reactions occur, the daily trend of the O_x_ variable is constant because O_3_ and NO_2_ have a symmetrical behavior strictly depending on the solar radiation, i.e. high ozone levels in the early morning with NO_2_ removal and opposite trend in the rest of the morning. During smog photochemical episodes higher levels of HCHO (Figure 7) and PAN are found: simultaneously, the various reactions cause an ozone accumulation and the relative sum of ozone and NO_2_ is not constant during the day. The behaviors of O_x_ and HNO_2_ can be considered an evidence of the occurrence of a smog photochemical episode (Figure 7).

In order to foresee photochemical pollution prevention, it is important to evaluate the relation between ozone and its precursors (VOCs and NO_x_). This evaluation has been always performed by mathematical models. In this approach we have considered the daily ratios of VOCs/NO_x_ determined in downtown Rome: they range between 1.3 and 5.5. About the VOC reactivity, from Table 2 it is possible to evidence that the olefin fraction is prevalent in the VOC composition (about 40%) and consequently plays an important and interesting role in the atmospheric chemical and photochemical reactions [26] with the aromatic fraction (about 15% of the total VOCs).

## Experimental

Measurements of PAN were carried out by means of a gas-chromatography (Carlo Erba Instruments, Milan, Italy). An electron capture detector (ECD) equipped with a ^63^Ni-foil of 10 µCi was used a glass tube (length 30 cm, i.d. 2 mm) packed with 10% Carbonwax on Chromosorb 80/100 mesh served with a column. Carrier-gas was nitrogen (purity of 99.99%). The flow-rate through the column was 20 mL min^-1^. The temperature of the GC oven was kept at 35°C, whereas the detector’s temperature was 100°C. An external pump (flow-rate 800 mL min^-1^) supplied the GC with ambient air, and every 15 min air samples (sampling volume of 2 mL) were automatically injected into the GC system through a 4-port valve regulated by pressurized air: the PAN retention time is 2.35 min whereas the detection limit is 0.001 ppb. Data were recorded by a Shimadzu integrator.

To prepare small amount of PAN a mixture of 50 ppm isobutene and 5 ppm NO_2_ diluted in synthetic air was undergone to irradiation by vapor Hg lamp [27].

Ozone and NO_2_ have been measured by means of a Differential Optical Absorption Spectrometry (DOAS, Opsis, Sweden) based on the Lambert-Beer’s law [28]. For describing the dynamics of the low boundary layer meaning the atmospheric stability/instability conditions was used the natural radioactivity by means of the β-radioactivity of short-lived decay products of Radon (SM200, Opsis) [29].

VOC concentrations have been measured by means of gas chromatograph POCP GC955 (Syntech Spectras, The Netherlands) equipped with two columns and three detectors. For the C_2_-C_5_ hydrocarbons an alumina column (15m×0.32 mm, 0.10 µm film thickness) (Alltech Ass. Inc., Illinois, USA) and two detectors flame ionization (FID) and photo ionization (PID) were used whereas for the C_6_-C_10_ hydrocarbons a column AT5 (15 m×0.32 mm, 0.10 µm film thickness) (Alltech Ass. Inc.) and a photo-ionization detector (PID).

The sampling site was located in downtown Rome (37 m a.s.l.; 41°54’N and 12°30’E; 2.7 million inhabitants), site characterized by high density of autovehicular traffic due to 2.5 millions among cars, motorcycles and bus (data from Automobile Club d’Italia) and domestic heating. The measurements covered 12-months from May 2007 to April 2008.

## Conclusions

The daily trends of ozone and PAN are reported and discussed together with the NO_2_ and HCHO behaviors in relationship with the concentrations of the natural radioactivity (radon) used as parameter for describing the dynamic of low atmospheric boundary layer. PAN has a low chemical reactivity and it represents a selective index of photochemical activity in atmosphere also because it is almost negligible the natural sources on its budget. Furthermore, PAN measurements are also important for investigating photochemical pollution transport phenomena.

For identifying the acute episodes of atmospheric photochemical pollution the PAN trends are shown and compared with those of the variable O_x_ (sum of NO_2_ and O_3_) describing specifically the evolution and the fate of oxidative processes due to atmospheric radical activity. At the same time, a VOC profile is reported and it is shown that the main contribution to the ozone formation comes from the olefinic fraction representing almost 40% of the total VOC amount. For this reason it is still an important issue to control the autovehicular emissions and to reduce and/or minimize the NO_x_ fractions in fuel.

Finally, as important part of the project, an analytical methodology based on GC-ECD analysis without sample enrichment and with high reliability and accuracy, was developed.

## Competing interests

The authors declare that they have no competing interests.

## Authors’ contributions

PA and MV set up the analytical procedure using GC-ECD. KM processed data and provided the comparison with other literature. PA and MV coordinated the study. PA edited the text and prepared the final draft of the paper. All the authors have read and approved the final manuscript.
